# Carbolic Acid in Cutaneous Disease

**Published:** 1868-12

**Authors:** 


Carbolic Acid in Cutaneous Disease.—Dr. F. P. Mann calls the attention of the profession to the efficacy of carbolic acid in the treatment of diseases of the skin, particularly those which are known to depend upon or are accompanied by the development of fungi. He reports three cases: one of chronic eczema, one of impetigo, and one of psoriasis. In the former case the incrustations covered the head and entire trunk and limbs. The secretions were intensely acid. By a course of alkaline treatment the improvement was rapid, but fresh groups of eczema pimples continuing to be re-produced, carbolic acid, of the strength of ʒ ss. to water ℥ iv, was applied three times a day. The effect was immediate, and the vesicles disappeared promptly. In the cases of psoriasis, the carbolic acid was applied in conjunction with the administration of Donovan's solution. The disease soon yielded to the treatment.—*N. Y. Medical Journal.*

